# Prolonged Cold Ischemic Time Before Normothermic Machine Perfusion Accentuates Postreperfusion Hepatocellular Injury

**DOI:** 10.1097/TXD.0000000000001943

**Published:** 2026-04-21

**Authors:** Shaheed Merani, Kennedy Scheele, Lance Fristoe, Maddie Cloonan, Sarah Uhm, Marian Urban, Kurt W. Fisher, Alan N. Langnas, Matthew T. Andrews

**Affiliations:** 1 Department of Surgery, University of Nebraska Medical Center, Omaha, NE.; 2 Department of Pathology, Microbiology, and Immunology, University of Nebraska Medical Center, Omaha, NE.; 3 School of Natural Resources, University of Nebraska Lincoln, Lincoln, NE.

## Abstract

**Background.:**

Prolonged cold ischemic time (CIT) before liver transplantation with static cold storage preservation is associated with higher rates of ischemia-related complications. With the introduction of normothermic machine perfusion (NMP), the overall incidence of early allograft dysfunction and biliary complications is lower. NMP can be used as a device-to-donor approach (with short CIT) or back-to-base model which is typically associated with longer CIT. It is unknown if NMP can rehabilitate organs that have suffered from prolonged CIT.

**Methods.:**

Livers from a Yorkshire cross-bred pig donation after circulatory death model were recovered using conventional in situ flush with University of Wisconsin solution after systemic heparinization, followed by static cold storage in University of Wisconsin solution at 4 °C for short (2 h), long (24 h), or extended (48 h) periods of CIT before ex situ NMP. Visual appearance of the liver, perfusion hemodynamics including pressure and flow, as well as perfusate concentration of lactate, aspartate aminotransferase, and alanine aminotransferase, bile production, and histological evaluation of liver biopsies stained with hematoxylin and eosin and caspase-3 were compared between groups during NMP.

**Results.:**

In addition to gross visual differences in the heterogeneity of livers with long or extended CIT compared with short CIT, the NMP perfusate from livers with extended CIT demonstrated an elevation in lactate that did not decrease with time. Although livers with long CIT showed a temporal reduction in NMP perfusate lactate concentration similar to those with short CIT, there was associated hepatocellular injury in the long CIT group manifested in the form of higher aspartate aminotransferase and alanine aminotransferase in perfusate, and increased frequency of caspase-3 positive cells indicating increased apoptosis.

**Conclusions.:**

While NMP offers improved clinical outcomes post-liver transplantation, the application of NMP in back-to-base format with prolonged CIT before NMP requires further evaluation to understand the impact of ischemia-reperfusion injury.

## BACKGROUND

Prolonged cold ischemic time (CIT) before liver transplantation using static cold storage (SCS) for organ preservation before transplantation is associated with higher rates of primary non-function (PNF),^1^ biliary complications,^2^ and the need for retransplantation.^3^ Specifically, CIT of >12 h has been observed to yield a 7% PNF rate (versus 0.4% PNF with CIT <12 h).^1^ CIT of >18 h is associated with a higher rate of graft loss and retransplantation.^3^ Increased rate of biliary strictures is observed in liver transplant using donation after circulatory death (DCD) donors with longer cold time.^2^ Increased postreperfusion syndrome (PRS) is observed following transplantation with longer CIT.^4^ These post-transplant clinical phenomena associated with prolonged CIT in SCS are believed to be manifestations of ischemia-reperfusion injury (IRI).

Normothermic machine perfusion (NMP) is a preservation and prognostication platform which involves ex situ continuous blood-based perfusion of intended liver allografts using oxygenated and heparinized blood along with nutrients and bile salts. When NMP is used for liver allograft management, the overall incidence of post-transplant graft IRI-related complications is significantly reduced.^5,6^ NMP reduces post-reperfusion injury and allograft cellular dysfunction when compared with SCS preservation, including mitigation of early allograft dysfunction (EAD), PRS, and ischemic biliary complications.^5,6^ Adoption of NMP by transplant centers in the United States has increased utilization of donors with higher donor risk index as well as DCD donor livers with higher age and national sharing.^7^ Although the reasons for this shift are not fully defined, it may be attributed to the rising expertise of transplant professionals in managing grafts on NMP, a platform where functionally active organs are amenable to rigorous biochemical and visual assessment. Additionally, NMP allows for longer preservation time to allow for organ allocation and transplantation logistics—with certain reports demonstrating liver preservation for up to 38 h with successful post-transplant outcomes.^8^ NMP may offer opportunities to reduce immunometabolic injury, particularly relevant to donor livers with pre-existing steatosis or advanced age. It is hypothesized that this effect is through reduced proinflammatory state as a result of NMP, altering the innate immune response post-transplant.^9^ Finally, recent studies have demonstrated that NMP improves value-based outcomes and reduces overall resource utilization in clinical liver transplantation.^10^

Despite the major benefits of NMP, its deployment and management in clinical liver transplantation require optimization. For example, NMP can be used as a device-to-donor approach (with short CIT) or back-to-base model (in which liver allograft is stored on SCS for transport from the donor to the recipient hospital, and attached to NMP only once at the recipient hospital) which is inherently associated with longer CIT. Additionally, logistical factors or late allocations may result in longer CIT before NMP. Overall, the characteristics and degree of IRI during NMP with prolonged pre-NMP CIT are unknown.

At least 2 retrospective clinical studies have evaluated pre-NMP CIT on the impact of post-transplant outcomes,^11,12^ however both studies were predominantly DBD livers. Indirect evidence of the impact of even short changes in pre-NMP CIT as a result of the need for pre-NMP arterial reconstruction are associated with higher rates of ischemic cholangiopathy,^13^ however the retrospective nature of this clinical study without randomized control livers (no arterial reconstruction) makes it challenging to interpret if differences are because of longer pre-NMP CIT alone or confounded by the need for arterial reconstruction.

Direct intent-to-treat clinical comparisons characterizing the impact of pre-NMP CIT on DCD liver transplant outcomes are currently lacking. This knowledge gap is increasingly critical as logistical paradigms shift between device-to-donor (short CIT) and back-to-base (prolonged CIT) deployment models. While pivotal trials report a mean CIT before NMP of only 2–3 h,^5,6^ the biological consequences of exceeding this window remain poorly characterized. To address this, we used a controlled porcine DCD model rather than a discarded human liver model. While human data are emerging, discarded livers often harbor significant selection bias, representing a heterogeneous population with pre-existing pathologies and variable ischemic histories that confound the isolation of CIT as a discrete variable. In contrast, a porcine model allows for precise control of warm ischemic time and donor health, providing the mechanistic clarity required to interpret clinical findings. Conceptually, we used NMP as a controlled experimental platform to isolate the reperfusion phase of IRI. By focusing on ex situ normothermic perfusion, we enable high-resolution serial assessment of hepatocellular injury and apoptosis, providing a reperfusion phase evaluation free from the confounding systemic immune responses and surgical variables inherent in a transplant recipient.

## MATERIALS AND METHODS

### Experimental Subject Livers

Livers were recovered from Yorkshire cross-bred porcine subjects using an established DCD model of organ recovery.^14^ Under isoflurane general anesthesia, after intravenous administration of heparin at 300 units/kg, and 20-min period of total warm ischemic time, livers were recovered using a super rapid recovery technique with abdominal aortic cannulation for administration of in situ cold (4 °C) University of Wisconsin (UW) solution flush and descending thoracic aorta cross clamp. Portal vein (PV) and hepatic artery (HA) were isolated and maintained during the surgical recovery. Autologous heparinized blood for subsequent perfusion experiments was obtained during these procedures.

### Institutional Review

Approval for all animal research was granted by the Institutional Animal Care and Use Committee (IACUC Protocol # 19-133-12-FC) at the University of Nebraska Medical Center.

### Static Cold Storage

**FIGURE 1.**
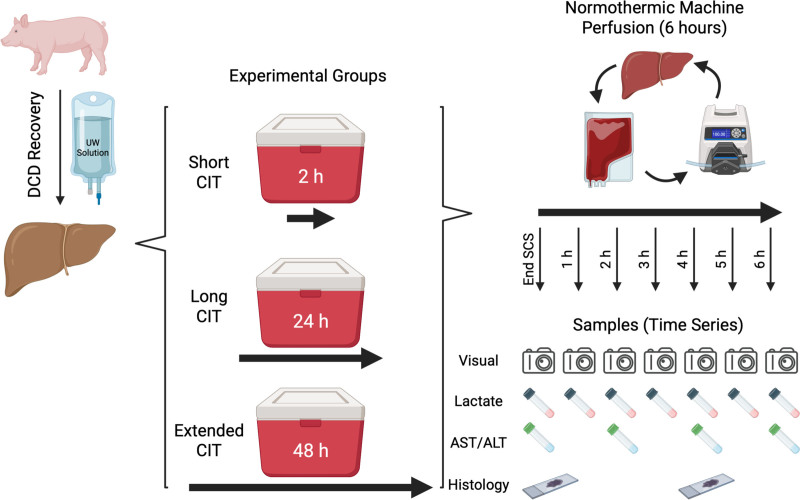
Schematic of experimental design. Livers recovered from a DCD porcine model were stored in UW solution for either short CIT (2 h, n = 9), long CIT (24 h, n = 9), or extended CIT (48 h, n = 3), before NMP for 6 h. During NMP, serial evaluations with visual assessment (hourly), perfusate biochemical testing (every 2 h), and tissue biopsy for histology were performed (at the end of SCS and 4 h NMP). ALT, alanine aminotransferase; AST, aspartate aminotransferase; CIT, cold ischemic time; DCD, donation after circulatory death; NMP, normothermic machine perfusion; SCS, static cold storage; UW, University of Wisconsin.

Livers were stored submerged in UW solution SCS at 4 °C, and randomly assigned to either short (2 h), long (24 h), or extended (48 h) periods of CIT before ex situ reperfusion using NMP as demonstrated in the study schematic (Figure 1). Pre-NMP CIT groups were selected on the basis that, in our clinical experience, the device-to-donor model of NMP requires 2 h of pre-NMP CIT for the process of donor hepatectomy, back table preparation, and cannulation of the liver allograft. This pre-NMP is consistent in clinical trial^5^ and in retrospective clinical studies.^11^ The extended CIT (48 h) groups was used to simulate extreme injury, while long CIT group (24 h) was used to simulate a situation of late allocation or logistical challenges requiring extended transport time of liver to recipient hospital on ice.^15^

**FIGURE 2.**
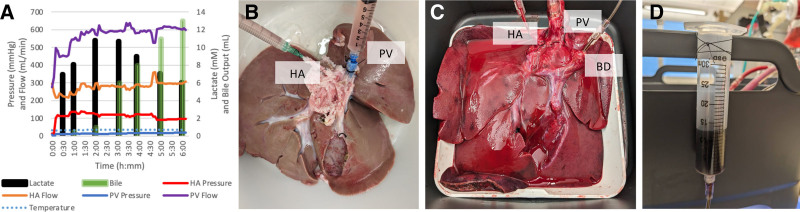
Representative example of porcine normothermic machine perfusion. Demonstrating perfusion measures (A), liver appearance following static cold storage (B), after 6 h of normothermic perfusion (C), and bile production (D). BD, bile duct; HA, hepatic artery; PV, portal vein.

At the end of the designated SCS period, and with the liver maintained cold, the PV and HA were cannulated for NMP, suture ligated in place, tested for vascular leaks, and then immediately before machine perfusion were flushed with cold saline solution to remove UW solution from the hepatic vasculature. The bile duct was cannulated for continuous monitoring of bile output during NMP, and the gallbladder was removed after ligating the cystic duct (Figure 2).

### Normothermic Machine Perfusion

A custom-built NMP circuit was used (Figure 2), consisting of a hard-shell venous reservoir with an integrated hollow fiber polypropylene membrane oxygenator and heat exchanger (Terumo FX05, Terumo Corporation, Tokyo, Japan). Perfusate temperature was maintained at 36.5 °C using a circulating water bath (Polyscience Digital Temperature Controller, Niles, IL). Blood tubing (¼-inch diameter) was driven by a centrifugal pump (Harvard Apparatus, Holliston, MA) with AP40 pump head (Medtronic, Minneapolis, MN) configured to allow independent HA and PV perfusion. The liver was positioned in a bespoke organ chamber (Joseph Joseph Inc, New York, NY) to allow passive venous drainage into the reservoir. HA and PV flow were measured continuously in real-time using SonoFlow ultrasonic flow sensor and A-Sonotec adapter (IWorx, Dover, NH), and pressure using PCU-2000 pressure control unit (Milar, Pearland, TX). Perfusate temperature was measured using a BAT-12 multipurpose thermometer (Physitemp Instruments, Clifton, NJ). All electronic signals were acquired using PowerLabC hardware and recorded using LabChart software (ADInstruments, Colorado Springs, CO). After NMP was completed, these signals were used to calculate the time-weighted mean temperature and PV and HA pressure and flow were calculated for each subject.

The hard-shell reservoir primed with autologous heparinized blood at 2 mL per gram of liver weight, and the circuit was primed with 15 000 units of sodium heparin (McGuff Pharmaceuticals Inc, Santa Ana, CA). NMP was performed for 6 h, during which serial perfusate samples and tissue biopsies were performed as detailed below.

A centrifugal pump was used to generate a total perfusion flow rate of 1 mL/min/g of pre-NMP liver weight. The integrated hollow fiber membrane oxygenator with heat exchanger was manually adjusted to achieve an initial target Po_2_ >300 mm Hg and target perfusate temperature of 34 °C. Perfusate was oxygenated with carbogen gas to maintain physiologic oxygenation parameters. Perfusate was directed to the PV, with a manual shunt to regulate flow to the HA. Total pump flow was set at 1 mL/min/g of liver mass with clamp adjustments applied, diverting blood from the PV toward the HA to achieve target PV flow of 55%–75%.

Continuous syringe pump infusions were initiated into the perfusate including Clinimix E TPN (4.25% Amino acid/10% Dextrose) (Baxter International Inc., Deerfield, IL) at 30 mL/h, epoprostenol sodium (Sun Pharma, Princeton, NJ) at 10 µg/h, and sodium taurocholate hydrate (Fisher Scientific, Waltham, MA) at 60 mg/h. Sodium Bicarbonate 8.4% (McGuff Pharmaceuticals Inc) was titrated to maintain perfusate pH at ≥7.10.

### Measures of Hepatic Physiology, Function, and Injury During NMP

Visual appearance during NMP was observed in real-time and photographs taken hourly. Cumulative bile production was recorded on an hourly basis through passive drainage. Peripheral wedge tissue samples were taken for histology before and at time points during NMP. Perfusate samples were taken for laboratory analysis, including lactate and glucose concentrations, which were measured hourly (i-STAT; Abbott Point of Care, Princeton, NJ), and the liver enzymes aspartate aminotransferase (AST) and alanine aminotransferase (ALT) activity were analyzed in the clinical laboratory (University of Nebraska Medical Center, Core Laboratory).

### Histology

Tissues are processed using a Sakura Tissue-Tek VIP, beginning with a graded dehydration series of 70%, 95%, and 100% Flex Alcohol, followed by Xylene clearing. The samples are then infiltrated and embedded into blocks with Histoplast PE wax using a HistoCore Arcadia H & C. Sections are cut at 4 µm on a microtome and mounted on positively charged slides. Following an overnight air-drying period at room temperature, slides are baked at 60 °C for 1 h. Finally, automated hematoxylin and eosin (H&E) staining is performed on a Sakura Tissue-Tek Prisma & Glas system using StatLab Select Hematoxylin and Reserve Eosin Multichrome.

### Caspase-3 Staining

Formalin-fixed paraffin-embedded hepatic tissue samples taken at pre-NMP and 4 h after NMP initiation were processed for immunohistochemistry to detect cleaved caspase-3. Immunohistochemistry staining was performed using a Discovery Ultra advanced staining system (Roche Diagnostics, Ventana Medical Systems). Sections were deparaffinized using a mild detergent solution with vortex mixing at 69 °C for 24 min (VENTANA EZ Prep solution, cat. 950-102) and rinsed with Tris-based reaction buffer (pH 7.6; cat. 950-300). Antigen retrieval was performed using CC1 tris-borate-ethylenediaminetetraacetic acid buffer (pH 8.2) at 95 °C for 24 min (cat. 950-124). Sections were then treated with Discovery ChromoMap RUO Inhibitor for 8 min (cat. 760-159). Slides were incubated with anti-cleaved caspase-3 primary antibody (rabbit polyclonal, 1:200, cat. ab4051; ABCAM) at 37 °C for 32 min, followed by horseradish peroxidase-linked anti-rabbit secondary antibody (Discovery anti-rabbit HQ RTU, 37 °C, 16 min; cat. 760-4815) and then enzyme conjugate (Discovery anti-HQ horseradish peroxidase RTU, 37 °C, 16 min; cat. 760-4820). Visualization was achieved with Discovery DAB RUO chromogen (cat. 760-159), and slides were counterstained with hematoxylin (cat. 790-2208 & 760-2037). The stained slides were used to assess tissue-level indicators of apoptosis in the setting of IRI.

### Microscopy and Image Analysis

A single expert gastrointestinal pathologist (K.F/) reviewed H&E-stained slides while remaining blinded to treatment group. Degree of ischemia and reperfusion injury observed histologically was qualified descriptively, and then further quantified using the Suzuki score which is based on the degree of congestion, vacuolization, and necrosis (reported as the sum of 3 subscores each on a range from 0 to 4).^16^

Images of 3 random regions of caspase-3–stained slides were acquired at ×10 and ×40 magnification. Staining was used to evaluate cleaved caspase-3 across timepoints, consistent with our focus on biologically relevant quantification of caspase-3 positivity in this porcine NMP model. Image files were imported into QuPath (version 0.5.1) for quantitative analysis. Images were designated as brightfield, and stain separation was achieved using a hematoxylin-DAB color deconvolution matrix. Pixel size was calibrated to 0.06 µm at ×40 magnification and 0.25 µm at ×10.

The full image field was annotated for downstream analysis. Cell identification was performed using the PositiveCellDetection module with optical-density–based detection and background correction. Nuclei were segmented with a Gaussian sigma of 1.5 µm, minimum area 10 µm^2^, and maximum 400 µm^2^. Watershed postprocessing refined nuclear boundaries, and cell bodies were expanded by 5 µm to incorporate cytoplasmic staining. Classification thresholds were based on nuclear DAB optical density, using a base detection threshold of 0.1 and positivity thresholds at 0.2, 0.4, and 0.6. Caspase-3 expression was quantified as percent-positive cells per annotated region. Three technical replicates demonstrated consistent staining and scoring behavior, and mean values of technical replicates were compared across timepoints between groups.

### Statistical Analysis

Continuous variables were compared between groups using nonparametric statistical test (Kruskal–Wallis test for 1-way configurations, and Scheirer–Ray–Hare test for 2-way configurations). All statistical analyses were performed in RStudio (Version 2024.12.0 + 467, Posit Software, PBC) using the following packages: *base, tidyverse, finalfit, rcompanion, rmarkdown, and ggplot2*.

## RESULTS

### Cold Ischemia Time Does not Impact HA or PV Pressure and Flow

No appreciable difference in HA pressure, HA flow, PV pressure, or PV flow was noted between pre-NMP CIT groups (Table 1). Temperature was maintained in all groups, with a trend toward lower oxygen saturation later in perfusion which may be indicative of increased oxygen consumption because of liver injury in those with long and extended CIT.

**TABLE 1.** Perfusion characteristics during NMP

	Short CIT	Long CIT	Extended CIT	*P*
Subjects (n)	9	9	3	
CIT, h	2.0 (2.0–2.0)	24.0 (24.0–24.0)	48.0 (48.0–48.0)	<0.001
Liver weight, g	842.0 (723.0–926.0)	840.0 (728.0–1076.0)	807.0 (783.0–910.0)	0.881
Bile production, cc	14.0 (7.2–23.8)	13.0 (6.0–29.0)	12.0 (6.0–16.0)	0.652
Bile, cc/kg/h	4.1 (2.5–5.1)	2.0 (1.4–6.0)	2.0 (1.0–3.1)	0.477
HA pressure, mm Hg	108.0 (70.3–139.5)	125.0 (114.1–161.4)	125.7 (108.0–143.3)	0.140
PV pressure, mm Hg	15.5 (13.1–16.8)	19.2 (9.5–20.9)	6.8 (6.0–7.7)	0.123
HA flow, cc/min	168.3 (135.8–240.8)	224.7 (182.7–250.2)	238.3 (231.5–245.2)	0.683
PV flow, cc/min	623.5 (597.3–659.4)	506.5 (424.1–616.9)	544.2 (540.7–547.7)	0.414
Temperature, °C	32.8 (32.6–33.6)	34.0 (33.8–34.1)	32.9 (32.5–33.3)	0.051
Po_2_ at prime, mm Hg	471.0 (471.0–471.0)	327.0 (301.0–360.0)	447.0 (321.0–450.0)	0.256
Po_2_ at 30 min NMP	433.0 (427.0–488.5)	444.5 (286.5–511.2)	383.0 (234.0–421.5)	0.617
Po_2_ at 4 h NMP	422.0 (422.0–422.0)	259.0 (70.8–451.0)	117.0 (96.5–228.0)	0.683

Reported are median (interquartile range), and *P* values using the Kruskal–Wallis test to examine differences between groups. Pressure, flow, and temperature are reported as time-weighted means over the entire NMP procedure.

CIT, cold ischemic time; HA, hepatic artery; NMP, normothermic machine perfusion; PV, portal vein.

### Extended Cold Ischemia Time Results in Visually Heterogeneous and Poor Perfusion

**FIGURE 3.**
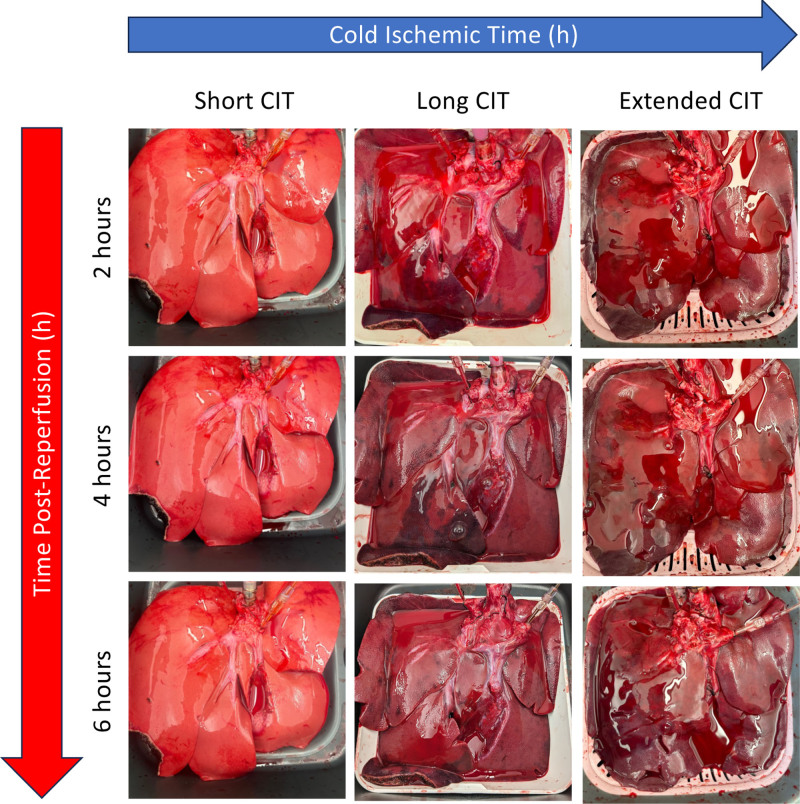
Visual appearance of liver during normothermic machine perfusion. Serial photographs of representative examples of a liver with short, long, and extended cold ischemic time during normothermic machine perfusion. Liver with short pre-NMP CIT demonstrates homogenous, well-perfused liver parenchyma, while those with long and extended time appear to be dark and heterogeneous. CIT, cold ischemic time; NMP, normothermic machine perfusion.

Livers with long and extended CIT were notably more heterogeneous after perfusion, a phenomenon that persisted after hours of NMP (Figure 3).

### Hepatic Function and Hepatocellular Injury

**FIGURE 4.**
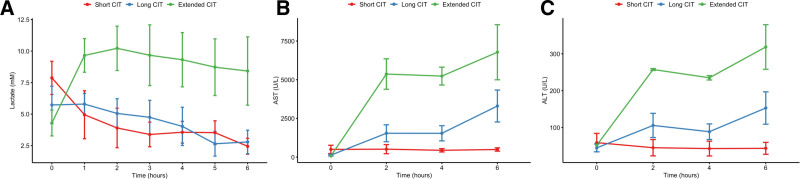
Perfusate biochemistry during NMP. Perfusate lactate (A) and liver enzymes (AST [B] and ALT [C]) during the course of normothermic machine perfusion. Group-based differences were observed in lactate, AST, and ALT (all *P* < 0.05, shown as mean ± SEM). ALT, alanine aminotransferase; AST, aspartate aminotransferase; CIT, cold ischemic time; NMP, normothermic machine perfusion; SEM, standard error of the mean.

Bile production during NMP was no different between CIT groups (Table 1). Livers with extended pre-NMP CIT demonstrated impaired lactate clearance; unlike the short and long CIT groups, which showed an early drop and maintenance of low lactate levels, the extended CIT group exhibited persistent lactate elevation (Figure 4).

Contributory to this finding was higher liver enzymes after reperfusion in those livers with extended CIT (Figure 4). Livers with long CIT did not show increased lactate compared to short CIT; however, they released incrementally higher AST and ALT, indicating a high degree of hepatocellular injury that worsened over the duration of NMP (Figure 4).

### Hepatocellular Injury Is Observed Histologically With Extended CIT Before NMP

**FIGURE 5.**
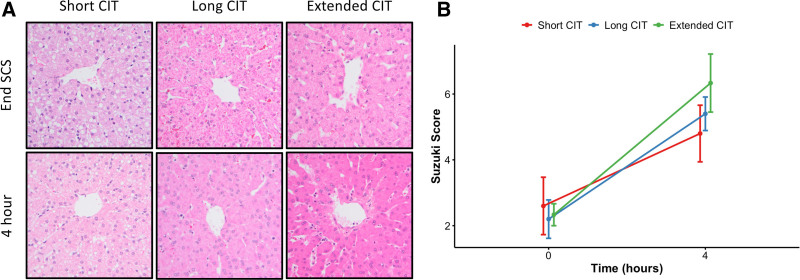
Histology of liver sections with variable CIT before NMP demonstrates increased ischemic change. Representative photomicrographs of H&E-stained slides (A) were taken at ×40 magnification of liver sections around central veins at the end of SCS (top row) and after 4 h of NMP (bottom row) for short (left), long (middle), and extended (right) CIT groups. Histologic characteristics of IRI were quantified using the Suzuki score (B), which showed a statistically significant time-dependent effect (*P* < 0.001, shown as mean ± SEM); however, the group-based effect did not reach statistical significance. CIT, cold ischemic time; H&E, hematoxylin and eosin; IRI, ischemia reperfusion injury; NMP, normothermic machine perfusion; SCS, static cold storage; SEM, standard error of the mean.

Microscopic evaluation of livers using H&E staining did not show appreciable damage in the short CIT group before or after NMP. All livers in the short CIT group were generally characterized as having hepatocytes with sharp nuclear detail without cytoplasm alterations surrounding intact central veins and sinusoids (Figure 5A, left column panels). However, both the long and extended CIT groups had easy-to-visualize histologic evidence of ischemic injury that was characterized by nuclei with faded staining and minimal nuclear detail, focus of hepatocytes without nuclei, and irregular cytoplasm clumping (Figure 5A, middle and right column panels). Furthermore, the extended CIT group showed additional signs of ischemic injury, such as numerous end-stage apoptotic single hepatocytes and loss of the endothelial integrity of the central vein with underlying apoptotic hepatocytes. H&E slides were additionally subject to evaluation using the Suzuki score to quantify histopathologic signs of IRI using criteria of congestion, vacuolation, and necrosis (Figure 5B).

**FIGURE 6.**
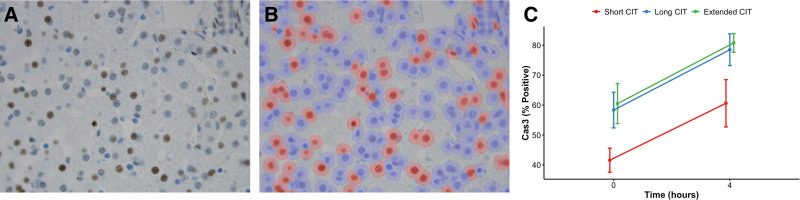
Prolonged CIT is associated with apoptotic hepatocellular injury. Representative photomicrograph of immunostaining for the apoptotic activation of caspase-3 (A), corresponding automated cell sorting (B) shows all cell detection with annotation for negative cells (blue) and positive cells (red) sorting. This was quantified (C), demonstrating a statistical time-dependent effect (*P* = 0.002, shown as mean ± SEM); however, the group-based effect did not reach statistical significance (*P* = 0.063). CIT, cold ischemic time; SEM, standard error of the mean.

To characterize hepatocytes that are committed to apoptosis, but not visible as apoptotic cells on H&E-stained slides, we performed immunostaining for activated caspase-3. The number of caspase-3–positive hepatocytes in the early stages of apoptosis was higher in long and extended CIT groups relative to short CIT (Figure 6). These microscopic findings of apoptosis in both long and extended CIT livers relative to short CIT livers are concordant with findings of elevated liver enzymes in the perfusate. Although no appreciable difference in the degree of hepatocellular apoptosis was observed between long and extended CIT livers, the graduated AST and ALT indicate a spectrum of injury with progressive CIT rather than an absolute tipping point for liver injury.

## DISCUSSION

Our data demonstrate that livers exposed to long CIT before NMP, although capable of clearing and maintaining low lactate concentrations, exhibit significantly higher liver enzyme release than those with short CIT and demonstrate a higher frequency of apoptosis, similar to the extended CIT group. While lactate clearance is a traditional clinical benchmark for NMP,^17^ our findings suggest that it may mask underlying hepatocellular injury. These results support the minimization of pre-NMP CIT to prevent cumulative injury,

IRI observed during NMP is closely associated with clinical outcomes such as EAD and PRS.^18,19^ As the back-to-base NMP model gains traction, particularly for the assessment and salvage of marginal liver allografts, as seen in the VITTAL^17^ and RESTORE^20^ clinical trials, understanding the compounding effect of cold ischemia is paramount. This is especially relevant in the United States, where recent organ allocation policy changes have introduced logistical inefficiencies, leading to increased CIT for allografts matched at higher offer sequences or accepted as expedited offers.^21^ While our data were generated using a model of standard DCD livers, the translational implications for extended criteria DCD grafts such as those with significant steatosis, advanced donor age, or pre-existing ischemic hepatitis are profound. These marginal grafts theoretically possess a lower threshold for ischemic tolerance; thus, the metabolic buffer provided by NMP may be significantly narrower if pre-NMP CIT is not strictly minimized.

The impact of pre-NMP CIT has been explored clinically with varying results. Bral et al^12^ compared back-to-base livers with locally recovered livers and found no significant differences in EAD or 6-mo graft survival. However, that study was limited by a predominantly DBD cohort (77%) and a relatively short CIT (mean 6 h) in the back-to-base group. In contrast, our study uses a controlled DCD model with fixed WIT and donor factors to isolate cold ischemia as an experimental variable. Furthermore, clinical trials often lack the serial biochemistry and post-NMP histology provided here. Our observation of increased caspase-3 activity and transaminase release suggests that while a graft may remain “clinically viable” by standard metrics, prolonged CIT induces a subclinical degree of injury that may affect long-term graft performance or biliary health, outcomes often underpowered in existing clinical reports.^11^

Although there is a paucity of clinical evidence that pre-NMP CIT results in increased IRI, there are preclinical rodent studies showing that 24 h of CIT results in higher lactate levels in rodent livers during subsequent machine perfusion. However, this research is notably limited since machine perfusion was performed using a non–blood-based system and that rodent livers are anatomically different from human livers.^22^ Our present study provides concordant findings, using the more clinically relevant large animal model of DCD livers with a blood-based perfusate.

Nostedt et al^23^ have previously demonstrated that the temperature of in situ flush solution (HTK) does not impact liver allograft viability, so long as NMP is subsequently used in a porcine DCD model. This study tests the hypothesis that core cooling results in liver injury. However, it did not evaluate the impact of CIT duration on IRI (livers were immediately placed on NMP without appreciable pre-NMP CIT). While our present research cannot isolate the contribution of cold exposure versus the ischemia time on the injury patterns observed during NMP in extended CIT livers, it does provide clarity that extended CIT before NMP is associated with hepatocellular injury.

Clinical strategies to minimize pre-NMP CIT include deployment of a device-to-donor or technical variations to minimize oxygen deprivation in static cold stored livers includes using oxygenated hypothermic perfusion during back table preparation of the liver allograft before NMP.^24^

We acknowledge several limitations. First, the sample size of the extended CIT (48 h) group was small, serving primarily as a “positive control” to define the absolute limits of the NMP platform. The study was primarily powered to detect differences between the clinically relevant 2- and 24-h cohorts. Second, the extended CIT (48 h) here is very long, and may not capture the more standard spectrum of CIT seen in a back-to-base model of NMP which would be expected to be in the 4- to 8-h range. However, the observation of CIT duration–associated apoptotic liver injury contributes to the current understanding of the limits of NMP. Additionally, we acknowledge that livers in the present report are only being evaluated during the NMP reperfusion (for reasons stated in Background) and not during subsequent liver transplantation. Therefore, the degree of recovery of hepatocellular injury observed during NMP following long and extended CIT in the post-transplantation phase has yet to be determined. Finally, we did not investigate whether extended NMP or specific perfusate additives may salvage livers experiencing IRI resulting from long or extended pre-NMP CIT. Despite this, our present study is contributory to the base of knowledge regarding pre-NMP CIT.

Further research is required to clarify the observations of this work. First, it is unknown if there is reversibility of the initial hepatocellular injury observed after long or extended CIT to allow for safe subsequent liver transplantation. This is particularly relevant since preclinical data relating to prolonged NMP is emerging,^18^ proposed to rehabilitate livers or facilitate allocation and placement. Second, it is unclear if the duration of NMP before liver transplant has the ability to rehabilitate injured liver allografts. Finally, the interaction between donor risk factors (such as steatosis or predonation transaminitis from ischemic hepatitis) and pre-NMP CIT is not yet characterized. This final consideration is specifically relevant to the proposition that NMP can be used as a platform for pharmacological interventions, such as defatting therapies.^25^

Our preclinical data demonstrate that prolonged CIT before NMP results in hepatocellular injury, with direct clinical relevance to the preferred modality for NMP deployment. Our data suggest that minimizing pre-NMP CIT (such as by using a device-to-donor rather than back-to-base configuration) results in reduced liver injury.
